# The effects of combination therapy with electroacupuncture and pelvic floor muscle exercise on stress urinary incontinence following radical prostatectomy: the protocol for a randomized controlled trial

**DOI:** 10.3389/fsurg.2025.1490210

**Published:** 2025-12-16

**Authors:** Xiaoyun Bi, Yuelai Chen, Ruoyu Wu, Weijia Gao, Qian Fan, Shengfei Wang, Jiahua Pan, Wei Xue, Qi-Xiang Song, Kangmin Tang

**Affiliations:** 1 Longhua Hospital, Shanghai University of Traditional Chinese Medicine, Shanghai, China; 2 Department of Urology, Ren Ji Hospital, Shanghai Jiao Tong University School of Medicine, Shanghai, China; 3 School of Acupuncture-Moxibustion and Tuina, Shanghai University of Traditional Chinese Medicine, Shanghai, China; 4 School of Rehabilitation Science, Shanghai University of Traditional Chinese Medicine, Shanghai, China

**Keywords:** electroacupuncture, pelvic floor muscle exercise, radical prostatectomy, stress urinary incontinence, urine leakage

## Abstract

**Background:**

Stress urinary incontinence (SUI) is one of the most common complications after radical prostatectomy (RP), affecting patients’ long-term quality of life. Electroacupuncture (EA) has been proven to be feasible in treating female SUI. However, considering the different pathogenesis of SUI between the two genders, it is essential to validate the efficacy of EA in male SUI after RP. So far, there has been no solid evidence confirming the benefit of EA in male SUI patients. Using the current study protocol, a prospective randomized controlled trial can be performed to reveal whether EA has additional benefits for accelerating SUI recovery after RP on the basis of pelvic floor muscle exercise (PFME). The outcomes from the proposed trial may provide key evidence for clinical guidance and practice to support the use of EA for post-RP SUI.

**Methods:**

In this prospective, randomized controlled trial, patients with SUI symptoms 6 weeks after RP will be recruited and randomly assigned to either treatment group (PFME plus EA) or control group (PFME plus placebo EA). Standard sacroiliac and abdominal acupoints will be selected for EA treatment. For placebo EA, blunt-tipped needles will be used to stab the skin surface at the same acupoints. Daily PFME is conducted with a unified protocol in both groups. The primary outcome is defined as the change from baseline in the 1-h pad test weight at week 6. The efficiency will be assessed using the intention-to-treat analysis by a statistician blinded to the interventions.

**Discussion:**

The study will provide a high level of evidence to justify the future application of EA by offering an alternative approach for the treatment of SUI after radical prostatectomy.

**Clinical Trial registration:**

ClinicalTrials.gov, identifier NCT05773716.

## 1 Introduction

The minimally invasive radical prostatectomy (RP) is the standard treatment for localized prostate cancer with tremendous benefits in intraoperative manipulation and postoperative recovery. However, it is still challenged by the incidence of stress urinary incontinence (SUI) after operation, which has no significant improvement compared with the traditional open radical retropubic prostatectomy (1, 2). There is a wide variation in the incidence rate of urinary incontinence, ranging from 4% to 40%, due to the different surgical techniques, definition of urinary incontinence, tumor staging, and epidemiological features across studies (3, 4). Despite multiple preservation and reconstruction techniques, postoperative SUI is still commonly reported due to the increasing overall surgical volume for prostate cancer (3). A great portion of SUI can be self-recovered taking up to 1–3 years, with the assistance of behavioral therapies, physiotherapies, medications, and devices (5). Surgical interventions are recommended for those with refractory and severe symptoms (5, 6). However, the use of implantation materials is frequently associated with the risks of infection, pain, tissue erosion, rejection, and surgical removal (5, 6).

Therefore, the exploration of non-invasive or minimally invasive treatment for male SUI, with high safety and effectiveness, has become a research hotspot. As an important part of traditional Chinese medicine, acupuncture treatment is promising to provide new ideas for the treatment of male patients with SUI after RP. Our initial clinical study has shown that periodic electroacupuncture (EA) treatment at Zhongliao and Huiyang acupoints can be valuable for female SUI patients, as evidenced by the continuously alleviated urinary leakage symptoms and quality of life (7). However, it is inappropriate to apply this evidence directly to male subjects, who have clearly different anatomical structures and incontinence mechanisms. With the aim to validate the true value of EA therapy on male SUI patients, we designed this prospective, randomized controlled trial, so as to provide high-level evidence for future clinical application of this modality. It is our intention to reveal whether EA offers additional benefits in accelerating recovery of SUI after RP on the basis of pelvic floor muscle exercise (PFME).

## 2 Methods

### 2.1 Study design

**Figure 1:**
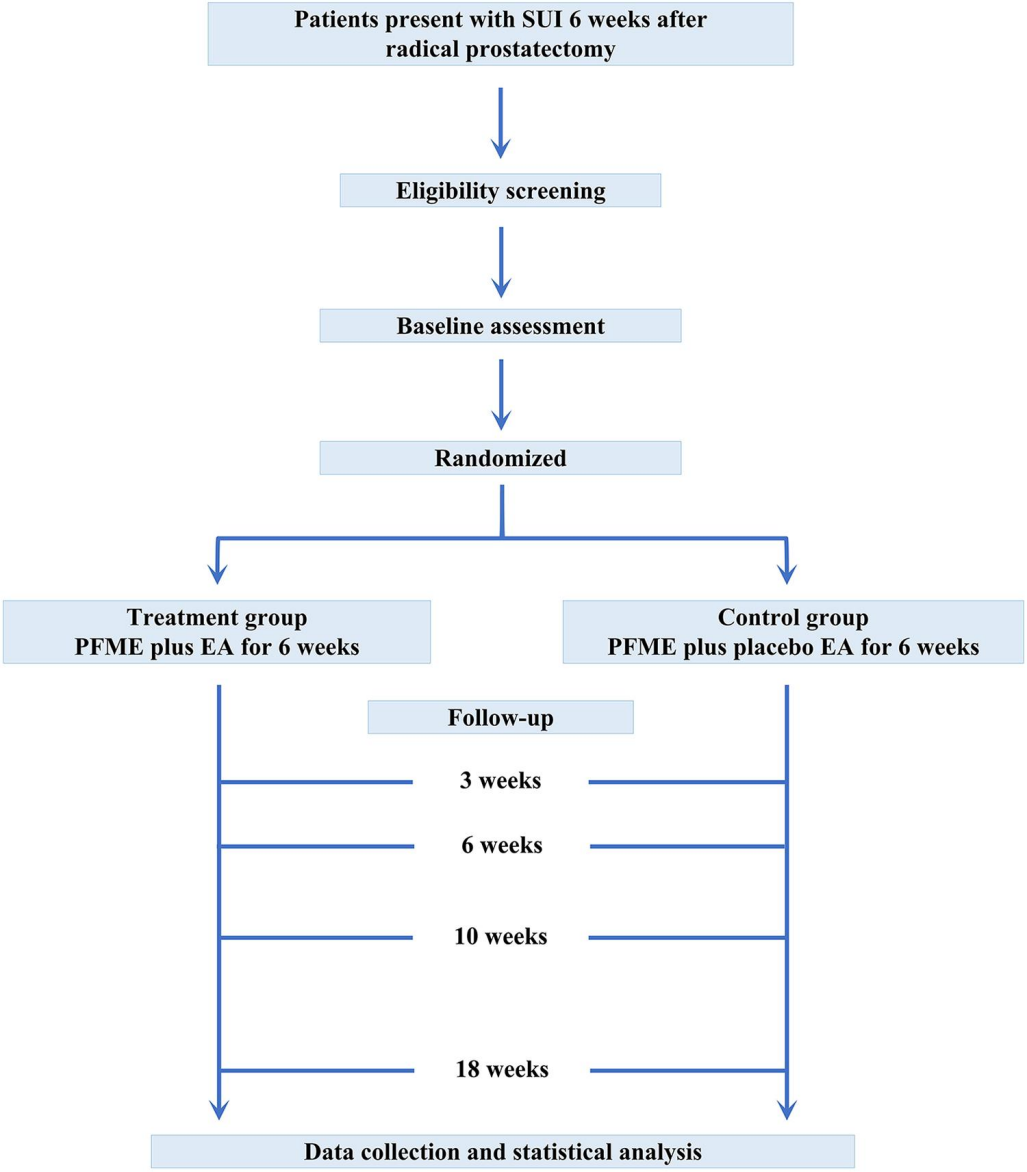
Study design. Male patients with persistent symptoms of stress urinary incontinence 6 weeks after radical prostatectomy will be recruited. After pre-admission screening and informed consent signing, they will be randomly assigned to the treatment group (PFME plus EA for 6 weeks) and the control group (PFME plus placebo EA for 6 weeks). After an eighteen-week follow-up, the data will be collected and analyzed.

This randomized, controlled trial will be conducted at Ren Ji Hospital, Shanghai Jiao Tong University School of Medicine, and LongHua Hospital, Shanghai University of Traditional Chinese Medicine. The study protocol has been approved by the Ethics Committee of Renji Hospital (LY2023-041-B) and Longhua Hospital (2023LCSY046) and is registered at ClinicalTrials.gov (NCT05773716). It has been prepared in accordance with the SPIRIT (Standard Protocol Items: Recommendations for Interventional Trials) 2013 guidelines. Any amendment to the protocol will be recorded and reported. After pre-admission screening and informed consent signing, they will be randomly assigned to the treatment group (PFME plus EA) and control group (PFME plus placebo EA). The assessments will be performed at baseline, and then at 3, 6, 10, and 18 weeks after treatment initiation. To ensure a comprehensive appraisal of the incontinence severity, 1-h pad test, the International Consultation on Incontinence Modular Questionnaire—Urinary Incontinence—Short Form (ICIQ-UI-SF), mean 24-h pad usage, and treatment response rate will be documented. Pelvic magnetic resonance imaging (MRI) will be performed to assess the changes in pelvic floor structure in relation to the improvement of incontinence symptoms. Despite the minimally invasive nature of EA therapy, the adverse events will still be carefully recorded based on the patients' reports. Data will be collected for analysis by a blinded specialist at the end of the trial. The flowchart of this study is shown in Figure 1.

### 2.2 Patient recruitment

This study will recruit male patients experiencing persistent SUI symptoms 6 weeks after RP from the inpatient and outpatient departments. Initially, trained researchers will screen potential participants through face-to-face evaluations based on the inclusion and exclusion criteria. Eligible patients will be enrolled at any time during the recruitment period.

Prior to randomization, each participant will provide written informed consent after receiving detailed study information. Key elements of the informed consent disclosure include: (1) random allocation of either EA plus PFME or placebo EA plus PFME; (2) a total time commitment of 18 weeks, comprising a 6-week intervention phase and 12 weeks of follow-up assessments; (3) potential risks such as minor bleeding, hematoma, or fainting, along with corresponding management measures; and (4) the voluntary nature of participation, including the right to withdraw at any time without affecting standard medical care.

Following consent, enrolled participants will be randomized and treated with the intervention by experienced acupuncturists. All personal information will be strictly confined to medical research purposes.

### 2.3 Inclusion and exclusion criteria

In general, this trial will recruit patients aged between 45 and 80, who present SUI symptoms 6 weeks after RP. The diagnostic criterion of SUI is in accordance with the International Continence Society standard, i.e., involuntary leakage of urine during an effort or exertion, or on sneezing or coughing. A 1-h pad test exceeding 1 g is used as the objective diagnostic criterion (7, 8).

To preclude confounding factors, patients with urinary incontinence before surgery, and urine leakage due to urgency incontinence, enuresis, chronic urinary retention, and fistula are excluded. Besides, those with a known history of pelvic radiation therapy, evidence of tumor recurrence or metastasis, and experience of other treatment for SUI (i.e., pelvic rehabilitation therapy, sling procedure, artificial sphincter, bulking injection, and proACT) are considered unsuitable for recruitment. To ensure safety, we also carefully screen out subjects with hemorrhagic tendency; the presence of ulcer, abscess, and skin infection at locations of acupoints; with a history of cardiac pacemaker, intravascular stent, and metal allergy; and with poor health condition due to the coexistence of acute systemic comorbidities. The detailed inclusion and exclusion criteria are listed in Table 1.

**Table 1:** The inclusion and exclusion criteria.

Inclusion criteria
• Patients present stress urinary incontinence 6 weeks after radical prostatectomy • Aged between 45 and 80 years • With a urine leakage amount over 1 g measured by the 1-h pad test • Participate voluntarily with signed the informed consent
Exclusion criteria
• The existence of stress urinary incontinence symptoms before the surgery • Urine leakage due to urgency incontinence, voiding dysfunction, nocturnal enuresis, and fistula • Known history of radiation therapy to the pelvic region • Evidence of tumor recurrence or metastasis • Those who have already undergone treatment for stress urinary incontinence • Patients with hemorrhagic disease or hemorrhagic tendency • The presence of ulcer, abscess, and skin infection at the locations of target acupoints • With histories of cardiac pacemaker, intravascular stent, and metal allergy • With poor health conditions due to the coexistence of acute comorbidities of the heart, brain, lung, and kidney • Those who refuse to sign the informed consent and are unable to comply with the study protocol

### 2.4 Randomization, allocation concealment and blinding

In this study, patients will be randomly assigned in a 1:1 ratio to either the treatment group or the control group. Participants, outcome assessors, and statisticians will remain blinded to group allocation throughout the trial. The allocation sequence will be generated by an independent statistician using a computer-generated random number list with variable block randomization through SAS software (version 9.4; SAS Institute Inc). The specific block sizes will be randomly varied and concealed from all personnel involved in patient recruitment and enrollment. This sequence will then be implemented using random allocation cards sealed in sequentially numbered, opaque envelopes by independent staff not involved in recruitment or intervention.

### 2.5 Interventions

#### 2.5.1 The acupoints selection

**Figure 2:**
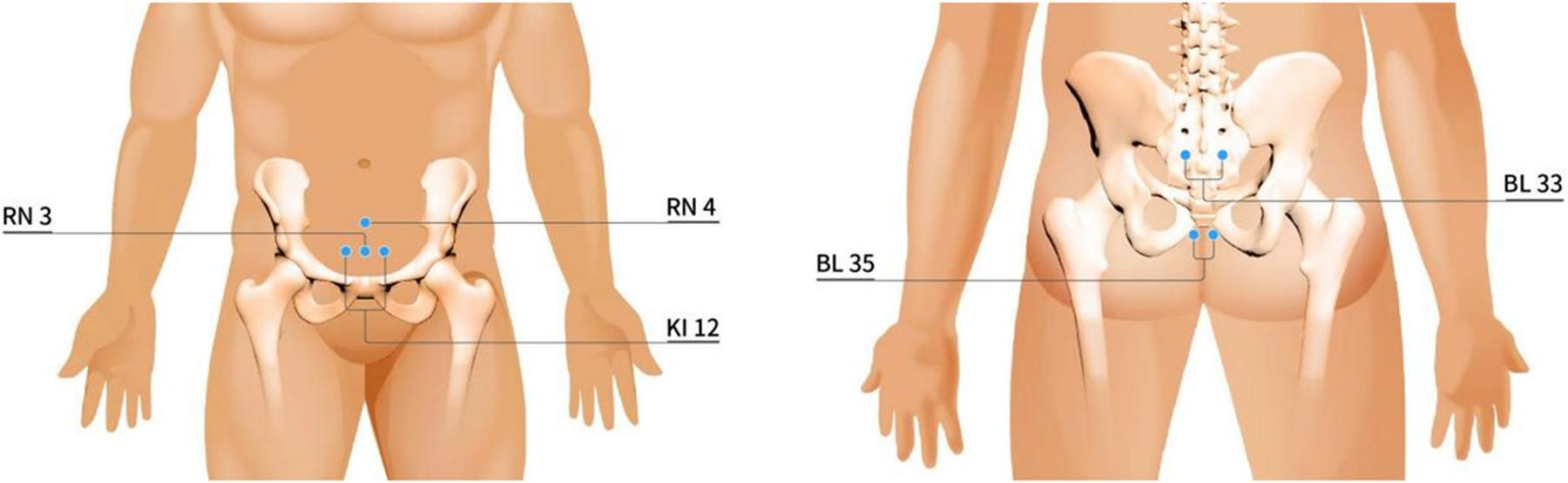
The acupoints selection. Five acupoints are selected referring to the National Standard of the People's Republic of China nomenclature and location of meridian points [GB/T 12346-2021]. RN4: the Guanyuan acupoint, on the anterior midline, 6 cm below the umbilicus; KI12: the Dahe acupoint, located 1 cm beside the anterior midline, 8 cm below the umbilicus; RN3: the Zhongji acupoint, on the anterior midline, 8 cm below the umbilicus; BL33: the Zhongliao acupoint, located at the third sacral foramen; BL35: the Huiyang acupoint, located 1 cm lateral to the extremity of the coccyx.

Both sacroiliac and abdominal acupoints will be selected referring to the National Standard of the People's Republic of China nomenclature and location of meridian points [GB/T 12346-2021]. In brief, with the patients in a prone position, the bilateral Zhongliao acupoints (BL33) are located at the third sacral foramen, and the bilateral Huiyang acupoints (BL35) are located about 1 cm lateral to the extremity of the coccyx (Figure 2). In a supine position, the Zhongji acupoint (RN3) is on the anterior midline, about 8 cm below the umbilicus; the Guanyuan acupoint (RN4) is on the anterior midline, about 6 cm below the umbilicus; the bilateral Dahe acupoints (KI12) are located about 1 cm beside the anterior midline, about 8 cm below the umbilicus.

#### 2.5.2 Procedures for needle insertion

For the treatment group (PFME plus EA), after disinfecting the skin of the sacroiliac region, a round sticky pad (about 1 cm in diameter and 0.5 cm in thickness) made from foam plastics will be pasted on the skin surface of each acupoint. The sticky pads are used to hold up the needles in place and to keep the patients blinded to the treatment, since for the control group, the skin is not penetrated. The 7.5 cm filiform needles will be inserted at an angle of 30–45 degrees in an inferomedial direction for BL33 and a superolateral direction for BL35 to a depth of 5–6 cm. The needles are manipulated slightly by twisting and lifting to achieve a sensation of soreness, numbness, distention, or heaviness in patients' perineum region (namely the reach of *deqi* in traditional Chinese medicine).

When needling the abdominal acupoints, RN3, RN4, and KI12 are accessed using a unified 7.5 cm filiform needle with an angle of 30–45 degrees, penetrating 4–5 cm in depth. The state of *deqi* is reached when the above-mentioned sensations occur at the bladder, perineum, and/or urethra areas.

For the control group (PFME plus placebo EA), the same sticky pad will be pasted on each acupoint following routine disinfection. The same acupoints will be punctured as those in the treatment group, except the use of blunt-tipped needles (identical in appearance compared with normal needles) to puncture through the fixed pad and to reach the skin surface. The needles will be lifted, thrusted, and twisted as described in the treatment group to create a tingling sensation, but without piercing the skin. The techniques of the placebo needle insertion have been well-reported in our previous publications (7, 9).

#### 2.5.3 The settings and treatment course of EA

In both groups, the needles will be clamped by electrodes that are connected to a pulse generator (Huatuo SDZ-EA, Suzhou, China). In consistency with our previous settings, continuous wave with frequency of 50 Hz will be used. The current intensity is gradually increased ranging from 1 mA to 5 mA, according to the patient's tolerance (7). For both groups, the treatment will be given every other day, three times a week, for continuous 6 weeks (18 sessions in total). For each session, both sacroiliac and abdominal acupoints will be punctured and retained for 15 min, separately.

#### 2.5.4 The protocol for PFME

To ensure the validity of exercise and adherence to the treatment, a trained therapist is available to guide the conduct of PFME throughout the trial. A PFME video will also be provided to each participant to standardize the performance of the exercise. In short, before training, the patients are required to empty their bladders and relax the whole body in a sitting or lying position. The contraction of the pelvic floor muscles is maintained for 2–6 s, followed by a relaxation for 2–6 s. This process is repeated 10 times per session, three sessions per day (in the morning, afternoon, and evening), and performed daily for 6 continuous weeks. The PFME program has been reported by several previous reports, and can be performed either on their own or in the office with assistance from the therapist (10–14). Each patient maintains a standardized exercise diary to log daily practice details. These diaries are reviewed weekly by the therapist during supervised sessions to discuss challenges, provide feedback, and calculate an objective adherence rate.

### 2.6 Outcome assessment

#### 2.6.1 General information

Data will be collected at the time of enrollment through structured questionnaires and electronic medical records, including key variables such as age, marital status, weight, height, educational level, smoking history, and comorbidities.

#### 2.6.2 Perioperative and surgical characteristics

These comprise the following key elements: the time interval from surgery to enrollment, prostate volume, detailed surgical specifics, e.g., surgical approach (single-port/multi-port), surgical technique (standard/“HOOD” technique), status of neurovascular preservation, extent of lymph node dissection, and post-operative pathological findings (including Gleason score and pathological staging).

#### 2.6.3 Primary outcome

The primary outcome indicator in this study is the difference in the 1-h pad test weight change from baseline to week 6 (7). In brief, the 1-h pad test begins with the patient emptying the bladder and wearing a pre-weighed, dry absorbent pad. During the 60-min testing period, the patient performs a series of standardized physical activities to provoke urinary leakage, including walking, climbing up and down stairs, repeated standing up from a seated position, coughing, and jogging in place. After finishing these activities, the pad is removed and re-weighed, and urine leakage volume is calculated as the weight difference (expressed in grams). This test is recommended by the International Continence Society as a standard tool to quantify urine loss (15).

#### 2.6.4 Secondary outcomes

The changes of pad weight from baseline during 1-h pad test will be compared between the two groups at 3, 10, and 18 weeks after treatment initiation. A validated ICIQ-UI-SF, containing 4 self-evaluation questions (i.e., frequency of incontinence, amount of leakage, impact of symptoms on quality of life, and timing of urinary leakage), will be used to assess the subjective symptoms at baseline, week 3, week 6, week 10, and week 18 (16). The mean 24-h pad usage will be assessed at 6 and 18 weeks after treatment initiation. This measure serves as a direct indicator of the patient's urine leakage experience and the associated disease burden in daily life (17). To establish a clear threshold for clinical efficacy, we have defined a “treatment response rate” as the reduction of ≥50% in urine leakage weight from baseline as measured by the 1-h pad test (7).

To determine whether EA could restore continence guarding mechanisms by alteration of pelvic structures, MRI will be performed (except for those with contraindications, such as metallic implants or psychological disorders) to evaluate the pelvic structural parameters, including membranous urethral length, urethral wall thickness, levator ani muscle thickness, and obturator internus muscle thickness before and after treatment. Pelvic MRI will be performed using a 3.0-T scanner with patients in the supine position. The imaging protocol will include an axial T1-weighted gradient echo sequence and high-resolution T2-weighted turbo spin-echo sequences in axial, coronal, and sagittal planes (TR: 4,000–6,000 ms, TE: 99–110 ms, slice thickness: 3 mm) (18).

Furthermore, any adverse event will be documented as reported by the patients or noticed by the researchers during treatment. The detailed study timeline is presented in Table 2.

**Table 2:** The study period.

Timepoint	Study period							
	Enrollment	Allocation	Intervention				Follow-up	
	Week-1	Week0	Week1	Week2	Week3	Week6	Week10	Week18
Enrollment:								
Inclusion/exclusion criteria	X							
Informed consent	X							
Randomization		X						
Interventions:								
[PFME plus EA]			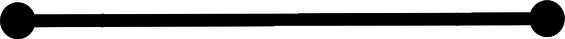					
[PFME plus placebo EA]			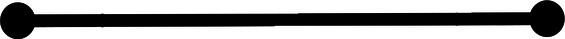					
Assessments:								
[Basic information]		X						
[Urinalysis]		X				X		
[Post-void residual^a^]		X				X		
[1-h pad test]		X			X	X	X	X
[ICIQ-UI-SF]		X			X	X	X	X
[24-h pad usage]		X				X		X
[Pelvic MRI^b^]		X				X		
[Adverse event]			X	X	X	X	X	X

EA, electroacupuncture; PFME, pelvic floor muscle exercise; ICIQ-UI-SF, international consultation on incontinence modular questionnaire—urinary incontinence—short form; MRI, magnetic resonance imaging.

a The post-void residual is measured by ultrasonography.

b The MRI can be waived when an absolute contraindication (metallic implants) is encountered.

#### 2.6.5 Safety evaluation

Adverse events (AEs) will be systematically monitored and documented throughout the trial. Specific anticipated treatment-related AEs include dizziness, fainting, subcutaneous hematoma, local infection, and needle breakage. Each AE will be meticulously recorded in the case report form, capturing the date of onset, severity (graded as mild, moderate, or severe), relationship to intervention (assessed as unrelated, likely related, or related), management measures implemented, and date of resolution.

All research acupuncturists will receive standardized training in both AE prevention and protocolized management. For any observed AE, immediate appropriate action will be taken. This may include cessation of needling, application of pressure to a hematoma, provision of rest for dizziness or fainting, or referral to a surgeon in the rare event of needle breakage. Any serious AEs will be reported to the safety committee without delay and managed actively. The frequency and proportion of participants experiencing any AE will be calculated and summarized by the investigators.

### 2.7 Statistical methods and sample size calculation

#### 2.7.1 Sample size calculation

In this study, the sample size was estimated according to the difference in the change in 1-h pad test weight from baseline to week 6. The calculation was informed by both previously reported evidence on EA for SUI (7, 9) and findings from our unpublished pilot study (*n* = 40) in post-RP SUI patients, which utilized identical interventions and involved a patient population with comparable baseline characteristics. The pilot data demonstrated a mean reduction of 58.1 g (from 83.1 g to 25.0 g) in the EA plus PFME group, compared to a mean reduction of 29.7 g (from 85.5 g to 55.8 g) in the placebo EA plus PFME group. Collectively, these sources supported an assumed mean between-group difference (effect size) of 28.4 g for the primary outcome, with a pooled standard deviation (SD) of 48.87 g.

Using PASS 2021 software (Version 21.0.3; NCSS, LLC), with a two-tailed significance level set at *α* = 0.05% and 80% statistical power to detect the aforementioned effect size (mean difference = 28.4 g, SD = 48.87 g), the minimum required sample size was calculated to be 96 participants (48 per group). To account for an anticipated dropout rate of 20%, the total number of patients to be recruited for the study was set at 120.

#### 2.7.2 Statistical analysis of treatment efficiency

All statistical analyses will be conducted using SPSS software (version 29.0, IBM Corp.) by a statistician blinded to group allocation and intervention details. The analyses will follow the intention-to-treat (ITT) principle, with per-protocol analysis conducted as a supplementary assessment. Missing data will be handled under the missing-at-random assumption using sequential regression multiple imputation.

Baseline demographic and clinical characteristics will be summarized using descriptive statistics. Continuous variables will be summarized as mean (standard deviation) for normally distributed data or median (interquartile range) for non-normally distributed data, and compared using independent samples *t*-tests or Mann–Whitney *U* tests, respectively. Categorical variables will be presented as frequency (percentage) and analyzed using chi-square or Fisher's exact tests, as appropriate.

The primary analysis will adjust for some covariates, such as the time interval from surgery to enrollment, to account for the potential influence. Repeatedly measured outcomes (1-h pad test weight, ICIQ-UI-SF scores, and 24-h pad usage) will be analyzed using mixed-effects models for repeated measures. Treatment response rates will be compared between groups using binary logistic regression or chi-square test. Additionally, changes in pelvic structural parameters measured by MRI will be analyzed using an appropriate mixed-effects model. The incidence of adverse events will be compared between groups using chi-square or Fisher's exact test.

To explore potential heterogeneity of treatment effects, pre-specified subgroup analyses will be conducted based on the following baseline factors: baseline severity of incontinence (stratified by 1-h pad test weight), patient age, and time interval from surgery to enrollment.

All statistical tests will be two-sided, with a *P*-value <0.05 considered statistically significant, except where adjusted for multiple comparisons.

### 2.8 Quality control, data management and monitoring

All investigators will undergo standardized training in the study protocol and standard operating procedures. Four licensed acupuncturists (YC, XB, QF, and KT) with a minimum of five years of clinical experience will receive additional rigorous training in standardized acupoint localization, manipulation techniques, and device operation. MRI measurements will be performed by two independent radiologists who have undergone standardized training in the predefined measurement protocols. To ensure data quality, inter-rater reliability will be rigorously quantified using the intraclass correlation coefficient.

Data collection will utilize specifically designed case report forms with an electronic tracking system. Our data management procedure requires independent dual-entry verification: one researcher enters data followed by confirmation by a second researcher, with all modifications systematically documented. Research assistants and monitors will regularly examine data quality and study progress. An independent monitoring committee, composed of experts in clinical practice, research methodology, and statistics with no direct involvement in the trial, will oversee trial conduct and ensure data integrity and safety through regular reviews conducted every six months.

## 3 Discussion

Even with the minimally invasive approaches and the emerging functional preservation/reconstruction techniques, SUI is still one of the most frequently reported post-prostatectomy complications jeopardizing patients' quality of life (3, 19–23). The current treatment modalities for postoperative SUI are usually chosen according to the patients' preference, symptom duration, and severity. The noninvasive physiotherapies (i.e., pelvic floor muscle exercise, biofeedback, electrostimulation, and magnetic therapy) are the first choice for patients with short-term postoperative incontinence or those with mild-to-moderate leakage (24, 25). For non-responders to initial conservative management, surgical interventions are usually warranted, such as urethral bulking injection, sling procedure, proACT, and artificial urinary sphincter (25, 26). Take the so-called “gold standard treatment” artificial urinary sphincter for example, although it has achieved a high cure rate in the systematic review, it is also associated with several peri- and postoperative morbidities, including but not limited to infection, erosion, mechanical failure, and urethral atrophy (27, 28). Therefore, an effective treatment with less invasion and high safety features is expected to be validated for male SUI.

In previous multicenter, randomized clinical trials, EA has been substantiated to be an effective approach for SUI in women. It is superior compared with the sham EA treatment in reducing pad weight and incontinence episodes, with very few adverse events (7). It also offers additional benefits to the conventional PFME in improving SUI symptoms and quality of life in female with SUI (9). However, due to the different anatomical features and mechanisms of SUI between women and men, it is still innovative and imperative to investigate whether the same treatment modality is applicable to male SUI patients. It is known that the incidence of female SUI mostly results from urethral hypermobility and insufficient supportive force of the pelvic floor muscles due to advancing age or long-term sequelae of vaginal delivery. Intrinsic sphincter deficiency, which may lead to a more disastrous urinary leakage symptom, is the major cause of SUI in about 13% of female patients, according to the urodynamic report from the VALUE study (29). In clear contrast, SUI in men is usually secondary to iatrogenic or traumatic events, and could have more integrated etiological factors. For example, for those who undergo radical prostatectomy, at least five contributors have been proposed, namely, reduction of functional urethral length, loss of anterior suspensory and posterior supportive structures, denervation injury, and direct external urethral sphincter damage (3). Therefore, it is challenging but necessary to justify the application of EA for male SUI patients after radical prostatectomy. Although some sporadic reports (mostly from Chinese domestic journals) have revealed that the EA might be beneficial for male SUI, the overall quality of evidence was considered low and inconclusive (30). Therefore, we proposed the protocol for a randomized, placebo-controlled trial, aiming to provide a high level of evidence to justify the use of EA in male SUI.

Unique from the previous study design focused on the comparisons between EA and sham EA treatment, we exclusively investigate the add-on effects of EA in addition to the conventional PFME in accelerating continence restoration after radical prostatectomy compared with the PFME alone (31). Besides, since studies have revealed a pattern of time-dependent continence restoration with a duration as long as 12–24 months after procedure, it is of great importance to study whether the combination of EA and PFME could facilitate the early regain of continence to improve quality of life and to avoid later application of invasive procedures (32, 33).

Previous studies have confirmed the efficacy of EA using sacral and abdominal acupoints for SUI. According to Traditional Chinese Medicine theory, post-RP SUI results from kidney qi deficiency and sinking of middle qi. Zhongliao and Huiyang from the Bladder Meridian can strengthen the kidney qi, while Guanyuan, Zhongji, and Dahe are selected to elevate the middle qi and consolidate the lower jiao (34–36). BL33 and BL35 overlie the S2-S4 sacral nerves, which govern bladder function. RN3, RN4, and KI12 align with the T12-L1 spinal segments, influencing the hypogastric plexus. EA acts through multiple synergistic mechanisms, including normalizing bladder reflexes, strengthening pelvic muscles, enhancing connective tissue support, and reducing local inflammation, to restore urethral function and continence (37, 38).

Several advantages can be summarized based on the current design. To start with, the choice of standard 1-h pad test results as the primary outcome instead of the simple questionnaires aims to provide an objective and rigorous evaluation of the true value of EA. Besides, the follow-up timepoints are selected to validate both the temporal effects of EA during 3 and 6 weeks, and sustained effects at 10 and 18 weeks after treatment completion. It is also worth mentioning that our design not only validates the effectiveness of EA but also investigates its influence on the modification of pelvic floor structures using MRI to reveal the potential mechanisms of treatment. Moreover, the stringent protocol for sham EA will be applied, in which a pragmatic placebo needle with a blunt tip will be used to create a sense of stabbing, but without penetrating the skin. The use of a sticky pad to maintain the angle of needles maximally ensures the blinded settings for both groups.

To date, the benefits of EA as an adjunct to PFME for male patients with SUI remain insufficiently studied. This research gap could lead to inconclusive and ambiguous clinical decision-making on this potentially effective intervention. The completion of a high-quality trial with the proposed study design will provide high-level evidence to justify the future application of EA by presenting an alternative approach for SUI after radical prostatectomy.
